# Real-world persistence with antiretroviral therapy for HIV in the United Kingdom: A multicentre retrospective cohort study

**DOI:** 10.1016/j.jinf.2017.01.012

**Published:** 2017-04

**Authors:** Joseph M. Lewis, Colette Smith, Adele Torkington, Craig Davies, Shazaad Ahmad, Andrew Tomkins, Jonathan Shaw, Margaret Kingston, Ghadeer Muqbill, Philip Hay, Larissa Mulka, Deborah Williams, Laura Waters, Nataliya Brima, Neal Marshall, Margaret Johnson, Mas Chaponda, Mark Nelson

**Affiliations:** a Royal Liverpool University Hospital, UK; b University College London, UK; c North Manchester General Hospital, UK; d Manchester Centre for Sexual Health, UK; e St Georges Hospital, London, UK; f Brighton and Sussex University Hospital, UK; g Mortimer Market Centre, London, UK; h Royal Free London, UK; i Chelsea and Westminster Hospital, London, UK; j Wellcome Trust Liverpool Glasgow Centre for Global Health Research, Liverpool, UK

**Keywords:** HIV, Antiretroviral therapy, ART, Persistence, Adherence

## Abstract

**Objectives:**

Persistence with an antiretroviral therapy (ART) regimen for HIV can be defined as the length of time a patient remains on therapy before stopping or switching. We aimed to describe ART persistence in treatment naïve patients starting therapy in the United Kingdom, and to describe differential persistence by treatment regimen.

**Methods:**

We performed a retrospective cohort study at eight UK centres of ART-naïve adults commencing ART between 2012 and 2015. Aggregate data were extracted from local treatment databases. Time to discontinuation was compared for different third agents and NRTI backbones using incidence rates.

**Results:**

1949 patients contributed data to the analysis. Rate of third agent change was 28 per 100 person-years of follow up [95% CI 26–31] and NRTI backbone change of 15 per 100 person-years of follow up [95% CI 14–17]). Rilpivirine, as co-formulated rilpivirine/tenofovir/emtricitabine had a significantly lower discontinuation rate than all other third agents and, excluding single tablet regimens, co-formulated tenofovir/emtricitabine had a significantly lower discontinuation rate than co-formulated abacavir/lamivudine. The reasons for discontinuation were not well recorded.

**Conclusions:**

Treatment discontinuation is not an uncommon event. Rilpivirine had a significantly lower discontinuation rate than other third agents and tenofovir/emtricitabine a lower rate than co-formulated abacavir/lamivudine.

## Introduction

The extent to which a patient takes medication in accordance with the expectations of the prescribing clinician can be quantified by a variety of methods. Early antiretroviral therapy (ART) for HIV necessitated extremely high levels of adherence,^1^ which can be defined as the degree of conformity to dose and dose interval.^2^ As a result of these requirements, studies exploring the consequences of poor adherence^1^, ^3^, ^4^, ^5^ as well as strategies for supporting compliance^6^, ^7^ to ART are well represented in the literature.

Persistence, however, refers to the length of time that a patient remains on therapy and can be defined as the duration of time from initiation to discontinuation.^2^ Persistency data for ART are lacking but can provide information on the comparative effectiveness, durability and tolerability of modern ART regimens in real-world patient populations. Differential persistence across different patient populations or according to patient characteristics can provide data to inform individualisation of treatment. Therefore, we performed a study to describe persistence with different ART regimens for treatment naïve patients starting ART in the United Kingdom (UK). We extracted data from established local treatment databases at eight UK HIV treatment centres to perform a retrospective cohort study comparing persistence across ART regimens in treatment naïve patients commencing therapy. In particular, we aimed to compare persistence across multiple tablet regimens (MTRs) by third agents and nucleo(t)side reverse transcriptase inhibitor (NRTI) backbone, and single tablet regimens (STRs).

## Methods

Eight UK specialist HIV treatment centres contributed data to this cohort; four in London, two in Manchester, one each in Liverpool and Brighton. Aggregate data were extracted from local treatment databases and/or clinical records. Inclusion criteria were: all treatment naïve people living with HIV commencing antiretroviral therapy between January 2012 and June 2015. Considered regimens were those within the British HIV association (BHIVA) guidelines at the time of the study and included in regional treatment guidelines: co-formulated tenofovir/emtricitabine (TDF/FTC) or abacavir/lamivudine (ABC/3TC) with ritonavir boosted darunavir (DRV/r), ritonavir-boosted atazanavir (ATV/r), efavirenz (EFV) or raltegravir (RAL), and co-formulated EFV/TDF/FTC or co-formulated rilpivirine (RPV)/TDF/FTC. All data were extracted in aggregate anonymised form and identifiable individual level data were not used. Patients were followed from date of ART start to the first date of treatment discontinuation, administrative censoring (June 2015) or last available viral load, and treatment discontinuation was defined to include stopping or switching therapy. Time to discontinuation was compared between ART regimens using incidence rates, with 95% confidence intervals calculated assuming a Poisson distribution when fewer than 20 evens, and with a Normal approximation otherwise. Statistical tests of difference in incidence rates, where carried out, used Poisson regression. As this study used only anonymised, aggregate data, ethical approval was not required.

## Results

A total of 2851 patients were initially identified; at two centres data were recorded by drug and not by regimen so for 902 patients taking EFV, TDF and FTC it was not possible to determine if they were taking STR or MTR. These patients were excluded from the analysis, leaving 1949 individuals. The median age was 37 (IQR 30–45) 1682/1949 [86%] were men, 1368/1949 [70%] men who have sex with men (MSM) and 1371/1949 [70%] of white ethnicity. Pre-treatment CD4 count data were missing for 138 patients; for the remainder, the median CD4 count was 343 (IQR 219–492) and 281/1811 [16%] patients had a CD4 count of less than 200 cells/mm^3^. There was missing pre-treatment viral load data for 75 patients; for the remainder 729/1874 [39%] had a viral load of greater than 100,000 copies/ml. The breakdown of the included regimens is shown in Table 1. Full baseline characteristics of the included patients are shown in Table 2.

**Figure 1:**
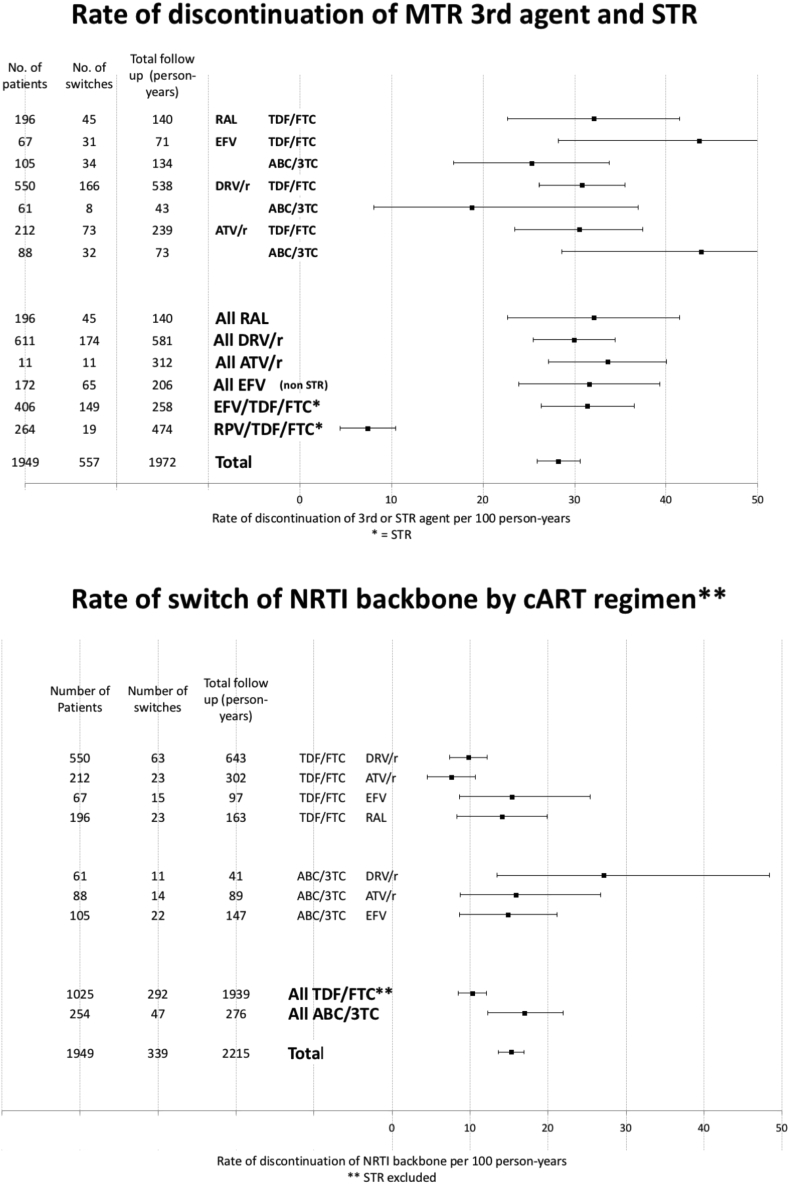
Discontinuation rates per person year of multiple tablet and single tablet regimen third agents (top), and NRTI backbone (bottom). EFV = efavirenz, TDF = tenofovir, FTC = emtricitabine, DRV/r = ritonavir boosted darunavir, ATV/r = ritonavir boosted atazanavir, RAL = raltegravir, 3TC = lamivudine, ABC = abacavir, STR = single tablet regimen, MTR = multiple tablet regimen.

During 2215 patient-years of follow-up – a mean follow up of 1.1 years per patient – 339 individuals changed the NRTI backbone, including as part of STR change (15 per 100 person-years [95% CI 14–17]) (Fig. 1). Comparing only MTR regimens, the rate of TDF/FTC discontinuation was significantly lower than ABC/3TC (10 [95% CI 9–12] vs 17 [12–22] per 100 person-years p < 0.01). During 1972 years of follow up – a mean follow up of 1.0 years per patient – third agent change was observed in 557 patients, including as part of STR change (28 per 100 person-year [95% CI 26–31]) (Fig. 1). The MTR third agents DRV/r, ATV/r, EFV, RAL had similar discontinuation rates (Fig. 1), as did EFV/TDF/FTC. However, RPV/TDF/FTC had a significantly lower rate (7 per 100 person-years [95% CI 4–11] p < 0.01 for comparison to all 3rd agent discontinuations.)

**Figure 2:**
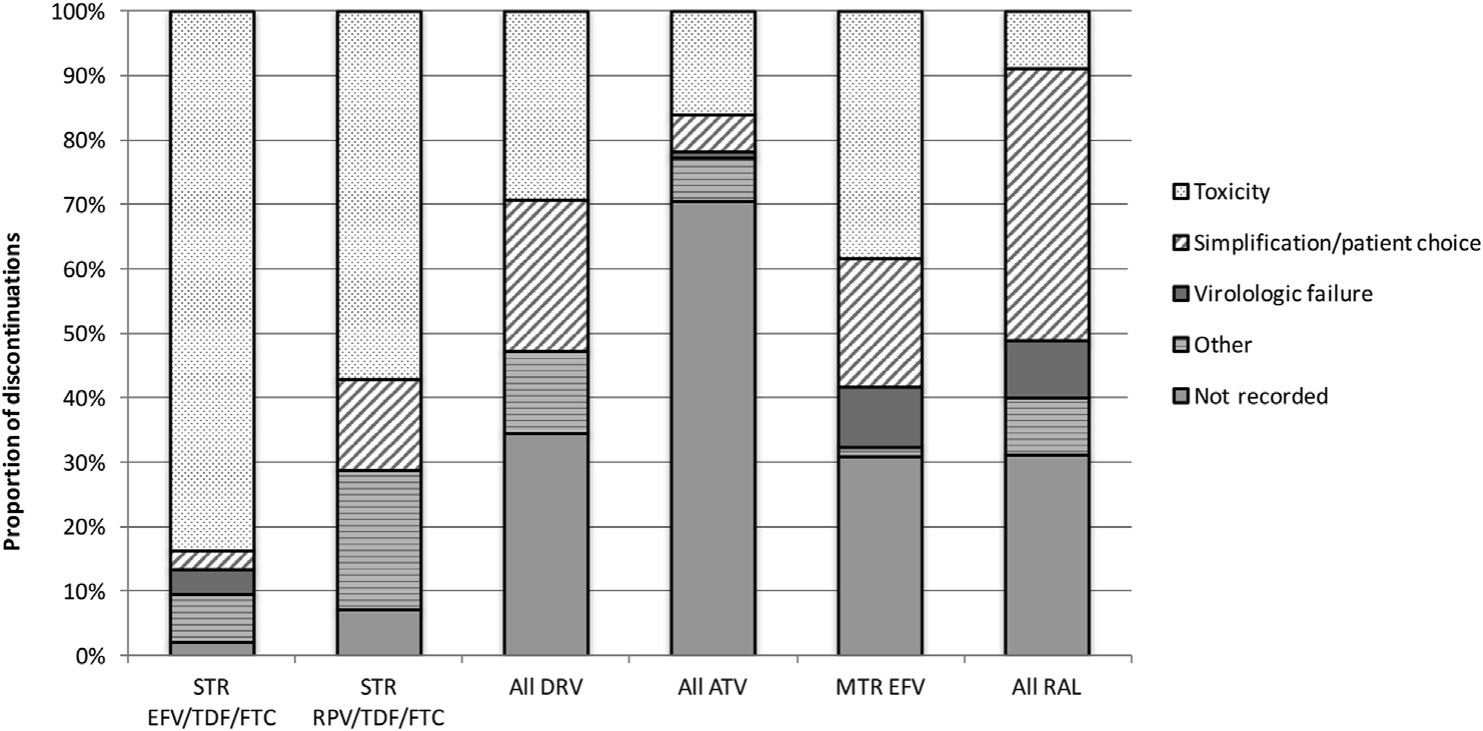
Reasons for discontinuation of multiple tablet regimen third agent and single tablet regimens. MTR = multiple tablet regimen, STR = single tablet regimen, ATP = co-formulated efavirenz/tenofovir/emtricitabine, EVA = co-formulated rilpivirine/tenofovir/emtricitabine, DRV = darunavir, ATV = atazanavir, EFV = efavirenz, RA = raltegravir.

**Figure 3:**
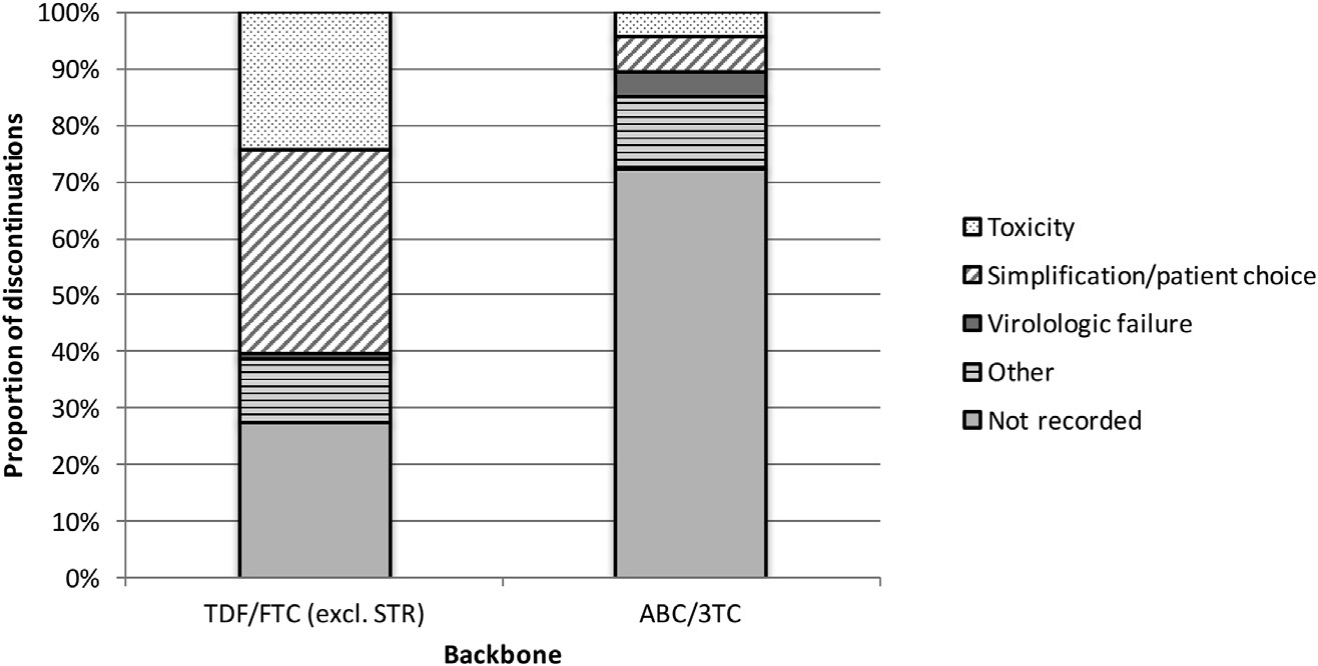
Reasons for discontinuation of NRTI backbone. STR = single tablet regimen, TDF = tenofovir, FTC = emtricitabine, ABC = abacavir, 3TC = lamivudine.

The reasons for discontinuations are shown in Figure 2, Figure 3. Of all MTR NRTI backbone discontinuations, the reason for discontinuation was not recorded in 68/171 [40%] of cases, and, of note, in the majority (34/47 [72%]) cases of ABC/3TC discontinuation. Of the remainder, 48/103 [47%] discontinuations were due to simplification or patient choice, 32/103 [31%] due to toxicity and 3/103 [3%] were due to virologic failure. Of all third agent and STR discontinuations, the reason for discontinuation was not recorded in 177/557 [32%] of cases. Of the remainder, 230/380 [61%] were due to toxicity, 85/380 [22%] were due to simplification or patient choice, and 17/380 [4%] due to virologic failure.

## Discussion

We describe the comparative persistence of antiretroviral therapy by initial regimen in nearly 2000 real-world people living with HIV in the UK in the UK. Patients enrolled in clinical studies may not be entirely reflective of the populations from which they are drawn; the large number of patients from multiple centres in our analysis provides a robust view of current day-to-day clinician and patient choices when initiating and discontinuing antiretroviral therapy.

It is possible to draw several conclusions from these data. Firstly, for patients in the UK, discontinuation of initial treatment is not an uncommon event. The discontinuation rate of third agent and NRTI backbone of 28 (95% CI 26–31) and 15 (95% CI 14–17) per hundred person-years respectively are comparable to those seen in other high-income settings: in an Italian cohort of 4052 patients starting ART between 2008 and 2014, discontinuation rates at 1,2 and 3 years were 26%,40% and 49%, respectively.^8^ These are similar to data from British Columbia, Canada, where the ART discontinuation rates of 2107 patients who initiated therapy between 2006 and 2010 were 36%, 47% and 53% at 1, 2 and 3 years,^9^ and Switzerland, where the rate of discontinuation of initial treatment in 1318 treatment-naïve patients was 42 per 100 person-years (95% CI 38–46).^10^ Older data from the UK are available from the UK CHIC cohort^11^; of 4583 patients initiating ART between 2000 and 2010, 15–19% discontinued therapy after one year (with exact proportion dependent on CD4 count); however, when patients who were also enrolled in the SPARTAC study of ART at seroconversion were excluded, the remaining 1746 patients had one-year discontinuation rates of 28–31%, highlighting the difference between clinical trial and real-world populations.

Secondly, in common with other studies, we found that where the reasons for discontinuation of therapy were recorded, they were overwhelmingly due to toxicity or simplification rather than virologic failure. However, the large proportion of records with missing data warrant caution in drawing any firm conclusions in this regard.

Thirdly, we observed significant differences in persistence with therapy across ART components. The rates of discontinuation of RPV as co-formulated RPV/TDF/FTC were considerably lower than all other considered regimens. Though the missing data on reasons for discontinuations make it difficult to draw firm conclusions, the fact that overall discontinuations in our cohort are driven by toxicity and simplification makes it likely that the very low rates seen in patients taking co-formulated RPV/TDF/FTC are driven by the favourable side effect profile and possibly the convenience of a STR. When examining MTR NRTI backbone we found a less striking, though still statistically significant, difference in rates of discontinuation of TDF/FTC and ABC/3TC, with lower rates of discontinuation of TDF/FTC. Rates of discontinuation of EFV/TDF/FTC were similar to MTR EFV.

Our study has a number of limitations. Most importantly, the data are retrospective and observational; decisions on choice of initial regimen and whether to discontinue are complex and result from a combination of clinician, patient, virus and drug characteristics. The persistence of co-formulated RPV/TDF/FTC may be due to the characteristics of the patients that clinicians in the UK are choosing to start on this regimen as well as the qualities of the regimen itself. In addition, decisions to switch therapy are based both upon the tolerability of the regimen and the availability of alternatives; the availability of newer STR containing elvitegravir or dolutegravir (which were infrequent in the local treatment databases and hence excluded from the analysis) could reduce the persistence of the regimens we have examined in the future. In addition, our data are aggregated; without individual-level data we are unable to perform a survival analysis to characterise the timing of treatment discontinuation, or to identify factors predictive of treatment discontinuation. This raises the possibility of selection bias of patients with more characteristics predictive of remaining on therapy towards RPV/TDF/FTC. We did not collect data on resistance patterns or HLA-B*5701 status, factors which will heavily influence initial ART regimen and the choice to discontinue.

In addition, mean follow up times were short, at around a year. The aggregate nature of the data means that the length of follow up of individual patients is not available, and so it is not possible to say how many patients were followed up for less than a year; however the fact that around a third of patients were recruited in the year prior to administrative censoring means that a significant minority of patients had follow up times of less than a year. This raises the possibility that, for some ART regimens, the discontinuation rate may be higher in the first year of therapy but a lower rate (for those remaining on therapy) for subsequent years on treatment; the lack of longer term follow up in these data do not allow this question to be addressed but caution should be exercised in extrapolating our results to long-term durability of treatment.

Despite these limitations, this study provides an insight into real life decisions with regard to initiation and discontinuation of ART in the UK, and the differential persistence of ART regimens in current use. These data may prove useful in guiding initial ART choice. Ultimately, a robust understanding of persistence and the determinants of persistence across available ART regimens will provide clinicians and patients with the tools needed to guide individualised ART, and maximise the chances of good outcomes for people living with HIV.

## Conflicts of interest

The authors report no conflicts of interest.

## Figures and Tables

**Table 1:** Included ARV regimens.

‘Third’ drug	NRTI backbone		All	CD4 count <200 cells/mm^3^	VL >100,000 copies/ml
Co-formulated EFV/TDF/FTC		n (%)	406 (20.8%)	86 (21.6%)	153 (21.0%)
Co-formulated RPV/TDF/FTC		n (%)	264 (13.6%)	19 (4.8%)	17 (2.3%)
DRV/r	TDF/FTC	n (%)	550 (28.2%)	149 (37.3%)	285 (39.1%)
DRV/r	3TC/ABC	n (%)	61 (3.1%)	15 (3.8%)	12 (1.7%)
ATV/r	TDF/FTC	n (%)	212 (10.9%)	32 (8.0%)	109 (15.0%)
ATV/r	3TC/ABC	n (%)	88 (4.5%)	17 (4.3%)	11 (1.5%)
EFV	TDF/FTC	n (%)	67 (3.4%)	23 (5.8%)	36 (4.9%)
EFV	3TC/ABC	n (%)	105 (5.4%)	14 (3.5%)	9 (1.2%)
RAL	TDF/FTC	n (%)	196 (10.1%)	44 (11.0%)	97 (13.3%)
**Total**			**1949 (100%)**	**399 (100%)**	**729 (100%)**

EFV = efavirenz, TDF = tenofovir, FTC = emtricitabine, DRV/r = ritonavir boosted darunavir, ATV/r = Ritonavir boosted atazanavir, RAL = raltegravir, 3TC = lamivudine, ABC = abacavir, NRTI = nucleoside reverse transcriptase, VL = viral load.

**Table 2:** Baseline characteristics of study participants.

		All	CD4 <200 cells/mm^3^	VL >100,000 copies/ml
Number		1949	399	729
Gender	Male n (%)	1682 (86.3%)	303 (75.9)	646 (88.6)
Age (years)	Median (IQR)	37 (30–45)	42 (34–49)	38 (31–46)
Risk for HIV acquisition	MSM	1368 (70.2%)	191 (47.9%)	519 (71.2%)
	Heterosexual	413 (21.2%)	169 (42.4%)	155 (21.3%)
	Other	168 (8.6%)	39 (9.8%)	55 (7.5%)
Ethnicity	White	1371 (70.4%)	209 (52.4%)	541 (74.2%)
	Black African	259 (13.3%)	110 (27.6%)	89 (12.2%)
	Black Caribbean	47 (2.4%)	13 (3.3%)	13 (1.8%)
	Other	271 (13.9%)	67 (16.8%)	86 (11.8%)
Viral load	Median (IQR) log cps/ml	4.8 (4.3–5.3)	5.3 (4.8–5.7)	5.5 (5.2–5.8)
	<1000 cps/ml	99 (5.1%)	13 (3.3%)	–
	1000–9999 cps/ml	230 (11.8%)	29 (7.3%)	–
	10,000–99,999 cps/ml	816 (41.9%)	83 (20.8%)	–
	≥100,000 cps/ml	729 (37.4%)	252 (63.2%)	729 (100.0%)
	Unknown	75 (3.9%)	22 (5.5%)	–
CD4 count (cells/mm^3^)	Median (IQR)	343 (219–492)	96 (38–151)	290 (120–419)
	<50	118 (6.1%)	118 (29.6%)	83 (11.4%)
	50–199	281 (14.4%)	281 (70.4%)	169 (23.2%)
	200–349	529 (27.1%)	–	175 (24.0%)
	350–499	450 (23.1%)	–	135 (18.5%)
	≥500	433 (22.2%)	–	121 (16.6%)
	Unknown	138 (7.1%)	–	46 (6.3%)
Year of starting ART	2012	694 (35.6%)	170 (42.6%)	252 (34.6%)
	2013	635 (32.6%)	118 (29.6%)	250 (34.3%)
	2014	570 (29.3%)	102 (25.6%)	211 (28.9%)
	2015	50 (2.6%)	9 (2.3%)	16 (2.2%)

VL = [HIV] viral load. ART = antiretroviral therapy.
